# Depression in Men and Women One Year Following Traumatic Brain Injury (TBI): A TBI Model Systems Study

**DOI:** 10.3389/fpsyg.2017.00634

**Published:** 2017-05-05

**Authors:** Sarah Lavoie, Samantha Sechrist, Nhung Quach, Reza Ehsanian, Thao Duong, Ian H. Gotlib, Linda Isaac

**Affiliations:** ^1^Rehabilitation Research Center, Santa Clara Valley Medical Center, San JoseCA, USA; ^2^Department of Neurosurgery, Stanford University, StanfordCA, USA; ^3^Department of Physical Medicine and Rehabilitation, Stanford University, StanfordCA, USA; ^4^Department of Psychology, Stanford University, StanfordCA, USA

**Keywords:** depression, mood, traumatic brain injury, concussion, gender

## Abstract

In the general population, females experience depression at significantly higher rates than males. Individuals with traumatic brain injury (TBI) are at substantially greater risk for depression compared to the overall population. Treatment of, and recovery from, TBI can be hindered by depression; comorbid TBI and depression can lead to adverse outcomes and negatively affect multiple aspects of individuals’ lives. Gender differences in depression following TBI are not well understood, and relevant empirical findings have been mixed. Utilizing the Patient Health Questionnaire-9 (PHQ-9) 1 year after TBI, we examined whether women would experience more severe depressive symptoms, and would endorse higher levels of depression within each category of depression severity, than would men. Interestingly, and contrary to our hypothesis, men and women reported mild depression at equal rates; PHQ-9 total scores were slightly lower in women than in men. Men and women did not differ significantly in any PHQ-9 depression severity category. Item analyses, yielded significant gender differences on the following items: greater concentration difficulties (cognitive problems) in men and more sleep disturbances (psychosomatic issues) in women per uncorrected two-sample *Z*-test for proportions analyses; however, these results were not significant after the family-wise Bonferroni correction. Our results indicate that, in contrast to the general population, mild depression in persons with moderate to severe TBI may not be gender-specific. These findings underscore the need for early identification, active screening, and depression treatment equally for men and women to improve emotional well-being, promote recovery, and enhance quality of life following TBI.

## Introduction

Depression is characterized by feelings of sadness, emptiness, hopelessness, and worthlessness. It can also be manifested by concentration problems, fatigue, and a loss of interest in previously enjoyed activities, as well as appetite, weight, and sleep disturbances; recurrent thoughts about death or suicidal ideation may also be present (American Psychiatric Association, 2013). Risk factors for depression include, experiencing adverse events during childhood such as emotional, physical, and sexual abuse (Chapman et al., 2004; American Psychiatric Association, 2013), memory and cognitive biases that influence emotion regulation (Foland-Ross and Gotlib, 2012; Kircanski et al., 2012), and genetic and physiological factors including a family history of depression, specifically among first-degree relatives, in addition to early onset or recurrent forms of depression (Sullivan et al., 2000; American Psychiatric Association, 2013).

Depression can affect multiple facets of life and cause clinically significant impairment or distress to individuals’ physical and emotional well-being, as well as to their social and professional life (American Psychiatric Association, 2013). Functional consequences can range from fairly mild, when depressive symptoms are undetected by others, to full impairment, such as the inability to address one’s own basic self-care needs (American Psychiatric Association, 2013). In the general medical setting, depressed individuals often experience higher levels of physical illness and pain and a diminished capacity for physical, social, and role functioning (American Psychiatric Association, 2013). Suicide is arguably the most serious consequence of depression (American Psychiatric Association, 2013); over half of all suicides transpire within the context of a mood disorder (Mann et al., 2005; Guillamondegui et al., 2011).

The prevalence rate of depression is estimated to be 8–10% in the general population (Guillamondegui et al., 2011). While epidemiologic findings elucidate that females are 70% more likely than males to experience depression (American Psychiatric Association, 2013; National Alliance on Mental Illness, 2016), clear gender differences in symptoms, course, response to treatment, and functional consequences have not been identified (American Psychiatric Association, 2013). Furthermore, some researchers have posited the Diagnostic Statistical Manual (DSM-5) (American Psychiatric Association, 2013), used to diagnose both psychiatric and neurological conditions, includes depressive symptoms that are more readily observed in female-specific depression (Weissman et al., 1984; Breslau et al., 1995; Piccinelli and Wilkinson, 2000; Kenneth et al., 2002).

Risk for depression among individuals with traumatic brain injury (TBI) is markedly higher than that seen in the general population (Scholten et al., 2016). Approximately 25–50% of persons with TBI will experience major depression within the first year after TBI (Scholten et al., 2016), and over 60% of persons are affected within 7 years after injury (Fann et al., 2009), reporting long-term struggles with a mood disorder. Importantly, major depression is associated with adverse outcomes for individuals with TBI (Barker-Collo et al., 2015; Bombardier et al., 2016; Moreno-Lopez et al., 2016), including social isolation, hostility and cognitive deficits (Mauri et al., 2014). Anxiety and addiction, along with other psychiatric conditions, may co-occur with depression in individuals with TBI (Gordon et al., 2006; Guillamondegui et al., 2011). Depression and comorbid psychiatric conditions can complicate depression screening, diagnosis, and treatment in a variety of ways, including depression being masked and ultimately left undiagnosed, and may interfere with an individual’s ability to comply and adhere to treatment (Gordon et al., 2006; Guillamondegui et al., 2011).

Little is known about the development and progression of depression following TBI (Bombardier et al., 2016), particularly among women (Oyesanya and Ward, 2016); this is due, in part, to the high symptom overlap (poor self-regulation, cognitive and inhibitory deficits) between TBI and depression (Schwarzbold et al., 2008). Despite the fact that depression is a frequently reported psychiatric condition following TBI, the extent to which depression influences long-term disability after TBI is not known (Crooks et al., 2007; Guillamondegui et al., 2011). Depression may be masked by cognitive changes, a flat affect, or other deficits commonly seen following TBI; these deficits may be attributed to the absence of post-trauma treatment progress when they are actually due to underlying depression (Guillamondegui et al., 2011). Risk factors for depression following TBI include structural changes in the brain due to injury (Cervos-Navarro and Lafuente, 1991; Xiao et al., 2015). Depression may also be a consequence of injury to the areas of the brain that manage emotions, in particular areas of the limbic system such as the amygdala (Blennow et al., 2012; Harmon et al., 2013). Changes in levels of neurotransmitter chemicals in the brain, which modulate emotions, have also been reported and can increase risk for depression following TBI (Blennow et al., 2012). Psychological response to injury may also elevate risk for depression in TBI (Lukow et al., 2015). Finally, depression can arise as individuals who have experienced TBI face new challenges in adapting to temporary or permanent disability, limitations, or changes in their professional, family, and community life (Lukow et al., 2015). Understanding differences in these factors between men and women is critical to early identification and treatment for depression in order to maximize recovery following TBI.

The Patient Health Questionnaire (PHQ-9) is a powerful clinical tool to study depression; it is able to detect a wide range of depression symptoms (Kroenke et al., 2001). Importantly, the PHQ-9 has been shown to be a sensitive instrument in identifying depression in individuals with TBI, especially in the hospital context (Dyer et al., 2016). The objective of this study was to assess depression, measured by the PHQ-9, in males and females 1 year following TBI. We hypothesized that, compared with males, females will report more symptoms of depression, operationalized as mean PHQ-9 total score, as well as a higher severity of depression within each of the five PHQ-9 depression severity categories, at a 1-year follow-up assessment.

## Materials and Methods

### Setting

This study was approved by the Santa Clara Valley Medical Center IRB Committee. All participants were admitted to the Rehabilitation Center of Santa Clara Valley Medical Center (SCVMC), a Level I Trauma hospital. The Rehabilitation Center is a specialty unit providing treatment to the most medically complex and acute traumatic brain-injured patients. This work was conducted at the Rehabilitation Research Center, SCVMC.

### Participants

Participants were from the Northern California TBI Model Systems of Care (TBIMS) database, a longitudinal study to assess long-term outcomes following TBI (Dahmer et al., 1993). All participants (1) met the TBIMS case definition for TBI (Dahmer et al., 1993); (2) met at least one of the criteria for moderate to severe TBI: post-traumatic amnesia > 24 h, trauma related intracranial neuroimaging abnormalities, loss of consciousness > 30 min, and/or Glasgow Coma Scale (GCS) < 13; (3) were age 16 years or older at the time of injury; and (4) sustained a TBI within California.

Inclusion criteria required that participants had a 1-year follow-up assessment completed between October 2007 and October 2013 (*n* = 238); these were the corresponding years that the PHQ-9 scores were obtained. Participants with a 1-year follow-up completed between October 2007 and October 2013 who had missing PHQ-9 data were excluded (*n* = 63); reasons for exclusions and missing data included lost to follow-up, refused follow-up, withdrew from study, incarcerated, expired, unavailable, and physically or cognitively unable to provide PHQ-9 responses. As described below, 175 individuals (*n* = 131 males; *n* = 44 females) met the inclusion criteria for analysis.

### Participant Characteristics

Demographic variables included sex, age, ethnicity, marital status, and employment status. Of the 238 participants who completed a 1-year follow-up between October 2007 and October 2013, 182 were male (75%) and 56 were female (25%); mean age was 32.38 (*SD* = 16.76). After excluding participants who were lost to follow-up (*n* = 7), refused (*n* = 2), withdrew (*n* = 1), incarcerated (*n* = 2), expired (*n* = 1), unavailable (*n* = 9), and physically or cognitively unable to provide PHQ-9 responses (*n* = 41), 131 males and 44 females (*n* = 175) were available for analysis. The racial and ethnic mix of the study population was White (*n* = 137), Black (*n* = 10), Hispanic (*n* = 65), Asian or Pacific Islander (*n* = 20), Native American (*n* = 1), and other (*n* = 5). Marital status was predominantly single (*n* = 149), followed by married (*n* = 61), divorced (*n* = 15), separated (*n* = 12), and other (*n* = 1). Years of education were: ≤8 (*n* = 7), 9–12 (*n* = 108), 13–16 (*n* = 109), and >16 (*n* = 14).

### Procedures

This study was carried out in accordance with the approval of the Institutional Review Board’s Research and Human Subjects Review Committee of SCVMC. All participants understood and provided informed consent to participate or, if unable, family or legal guardian understood and provided informed consent for the patient in accordance with the Declaration of Helsinki. Data regarding injury was collected via medical record abstraction by a research assistant. At 1-year post-injury, a research assistant conducted follow-up assessments for data collection.

### Measures

The PHQ-9 (Kroenke et al., 2001) is a self-reported 9-item measure of depression. Scores are totaled and are categorized into level of severity with higher scores indicating greater depressive symptoms (1–4: minimal depression, 5–9: mild depression, 10–14: moderate depression, 15–19: moderately severe depression, 20–27: severe depression). The PHQ-9 has good test-retest reliability (*r* = 0.84) and internal reliability (α = 0.89) (Kroenke et al., 2001). The PHQ-9 is valid and reliable in persons with TBI (Fann et al., 2005).

### Analyses

Demographic variables, as mentioned above, were obtained for all participants who completed a 1-year follow-up assessment between October 2007 and October 2013 (*n* = 238). The final sample for analyses (*n* = 175) was then divided into two groups on the basis of sex. For each group, mean PHQ-9 total scores were calculated. A two-tailed *t*-test was used to compare the average total PHQ-9 scores between the two groups, with alpha set at *p* < 0.05. The percentages of individuals in each category of depression severity was calculated for each group. Two-sample *Z*-tests for proportions were then used to compare the categories of male versus female within each category of depression. The percentage of individuals who endorsed each of the 9-items of depressive symptoms was calculated for each group to provide an item analysis assessment. Two-sample *Z*-tests for proportions were then used to compare the proportion of males versus females for each item.

Initial analysis was conducted using Microsoft Excel; a data analysis cross check was then performed using IBM SPSS Statistics 24. An independent sample *t*-test was used to cross check for mean PHQ-9 total scores, following an outlier and range check. Chi-squared tests were used to cross check all initial *Z*-tests run for depression severity categories and item analyses; the Bonferroni correction was applied to correct for any family-wise errors arising from multiple comparisons in the data analyses.

## Results

### PHQ-9 Total Scores

Both men and women reported experiencing mild levels of depression (PHQ-9 Total Score = 5–9). Total PHQ-9 scores in men (*M* = 6.04; *SD* = 6.26) were higher than total PHQ-9 scores in women (*M* = 5.27; *SD* = 5.58), but this difference was not statistically significant [*t*(173) = -0.72; *p* = 0.47].

### PHQ-9 Depression Severity Categories

A lower percentage of males (17.56%) than females (27.27%) reported experiencing no symptoms of depression, mild depressive symptoms (22.90% vs. 27.27%), and moderately severe depressive symptoms (6.11% vs. 6.82%); uncorrected two-sample *Z*-test for proportions analyses comparing males and females, however, revealed no statistically significant differences in any PHQ-9 depression severity category (|z|≥ 0.84; *p* ≥ 0.16) (see **Table 1**).

**Table 1:** Comparison of PHQ-9 depression severity categories.

Total score	Depression severity	Females	Males	*P*-value
0	No depression	27.27%	17.56%	0.17
1–4	Minimal depression	29.55%	36.64%	0.40
5–9	Mild depression	27.27%	22.90%	0.56
10–14	Moderate depression	6.82%	11.45%	0.38
15–19	Moderately severe depression	6.82%	6.11%	0.87
20-27	Severe depression	2.27%	5.34%	0.40

*Two-sample *Z*-test for proportions did not show significance at uncorrected *p* < 0.05. However, interesting trends emerged in the data.*

### PHQ-9 Item Analysis

The uncorrected item analysis yielded statistically significant sex differences for the following items: males endorsed “trouble concentrating on things, such as reading the newspaper or watching television” for “more than half the days” to a greater degree than did females (12.21% vs. 0%) (*z* = 2.43; *p* = 0.02), and females endorsed “trouble falling or staying asleep or sleeping too much” for “several days” to a greater degree than did males (31.82% vs. 16.79%) (*z* = -2.13; *p* = 0.03) (see **Table 2**). However, these results did not reach significance using Bonferroni corrected alpha of *p* = 0.0014.

**Table 2:** Item Analysis of PHQ-9.

Questions	Not at all (0)	Several days (1)	More than half the days (2)	Nearly every day (3)
(1) Little interest or pleasure in doing things	0.34	0.18	0.31	0.36
(2) Feeling down, depressed, or hopeless	0.96	0.52	0.98	0.40
(3) Trouble falling or staying asleep, or sleeping too much	0.41	0.03^∗^	0.76	0.17
(4) Feeling tired or having little energy	0.45	0.11	0.89	0.60
(5) Poor appetite or overeating	0.98	0.40	0.90	0.67
(6) Feeling bad about yourself – or that you are a failure or have let yourself or your family down	0.90	0.25	0.38	0.58
(7) Trouble concentrating on things, such as reading the newspaper or watching TV	0.15	0.91	0.02^∗^	0.99
(8) Moving or speaking so slowly that other people could have noticed. Or the opposite – being so fidgety or restless that you have been moving around a lot more than usual	0.08	0.76	0.27	0.18
(9) Thoughts that you would be better off dead, or of hurting yourself	0.72	0.87	0.07	0.31

*^∗^Two-sample *Z*-test for proportions showed significance at uncorrected *p* < 0.05.*

*Item 3: f > m; Item 7: m > f.*

*Family-wise Bonferroni correction was set at *p* < 0.0014.*

## Discussion

Individuals with TBI are at a significantly higher risk compared to the general population for developing both acute and chronic depression (Scholten et al., 2016), and for the emergence of various psychiatric disorders (Iverson et al., 2011; Brandel et al., 2016) such as anxiety and post-traumatic stress disorder (PTSD) (Zaninotto et al., 2016), and suicidal thoughts and behaviors (Fisher et al., 2016). Given the lethality concerns surrounding depression, raising awareness about the prevalence of depression in persons with TBI is especially important.

The present study of sex differences in TBI and depression is critical given recent findings from the Centers for Disease Control and Prevention (2016) indicating that the prevalence of women with mild to severe TBI, as measured by TBI-related Emergency Department visits in the U.S. among the general population, has risen by 49% between 2007 and 2010; rising rates among women have been attributed to more females experiencing falls, motor vehicle accidents, unintentional blunt traumas, and assaults (Centers for Disease Control and Prevention, 2016; Oyesanya and Ward, 2016). Importantly, depression rates are increasing in parallel to the rising rates of TBI (Oyesanya and Ward, 2016). Previous studies reveal that female survivors of TBI are at higher risk for developing depressive disorders than are male survivors (Iverson et al., 2011; Oyesanya and Ward, 2016; Scholten et al., 2016). Other research, however, suggests that there are no gender differences in outcomes post-TBI (Mushkudiani et al., 2007; Slewa-Younan et al., 2008; Renner et al., 2012), and still other findings indicate that males with TBI are at a higher risk for depression than are females (Sigurdardottir et al., 2013; Albrecht et al., 2015), particularly when comparing post-menopausal women with age-matched men (Davis et al., 2006). Investigators have found that younger women have better outcomes than do older women post-TBI; this may be attributable to the neuro-protective effects of hormones found in pre-menopausal women (Kirkness et al., 2004; Wagner et al., 2004; Ley et al., 2013). Despite conflicting results, researchers and clinicians alike are becoming aware of hormonal disturbances that often follow TBI (Ghigo et al., 2005; Schneider et al., 2011; Lauzier et al., 2014). Therefore, it is imperative for depression treatment care plans following TBI to be tailored to each gender specifically to address the physiological and hormonal differences between men and women who are affected by brain injury.

Investigators have suggested that TBI-related depression results from altered functional connectivity of a number of networks in the brain, including white matter abnormalities (Isaac et al., 2015), and neuroadaptions within the thalamus, insula, and subgenual cingulate cortex (Moreno-Lopez et al., 2016). Disruption to the neural circuitry between the limbic system and the prefrontal cortex, that results from diffuse axonal injury (Inglese et al., 2005; Silver et al., 2009), as well as from damage to the hippocampus, amygdala, and prefrontal regions of the brain (Harmon et al., 2013), has also been found to lead to mood disorders that develop as soon as a few weeks or months following the initial injury (Jorge and Starkstein, 2005). A number of other factors, including various adjustment issues and unique barriers that accompany TBI recovery (Moreno-Lopez et al., 2016) such as low levels of social support, being young at time of injury (Ouellet et al., 2009), and lack of hope (Oyesanya and Ward, 2016), may also contribute to depression emergence among the TBI population. Indeed, one of the most disabling symptoms of depression is lack of hope; depression, comorbid with TBI, is therefore a leading contributor to disability following TBI (Oyesanya and Ward, 2016). These contributing factors for depression may help explain why gender differences are not as prevalent among those with TBI compared with the general population. Both males and females with a TBI experience life-altering changes that directly impact their quality of life and productivity. Future research is needed to quantify the extent to which depression emergence post-TBI is a direct result of structural and neurochemical changes that occur in the brain following the injury versus due to the associated barriers in TBI recovery attributable to suffering a TBI.

Interestingly, unlike the general population, the mild depression noted among both males and females with TBI in our study population suggest that depression is not gender-specific. Gender differences may be seen, however, in the manifestation of depression symptoms. We demonstrated that a greater percentage of men reported concentration difficulties, where depression was experienced through cognitive symptoms, while a higher percentage of women indicated sleep disturbance issues, reflecting more psychosomatic symptoms of depression. These exploratory results are consistent with previous research demonstrating that depression symptoms are expressed differently in men and women; whereas men experience cognitive symptoms (Alexandrino-Silva et al., 2013), women are more affected psychosomatically (Alexandrino-Silva et al., 2013; Silverstein et al., 2013); however, our item analyses did not reach statistical significance using a strict Bonferroni correction. These findings warrant further study in order to better understand depression following TBI and its unique impact on each gender. Moreover, our results indicate the need for early identification, active screening, and treatment of mood disorders in both genders equally to improve emotional functioning, reduce disability, promote recovery, and enhance quality of life following TBI.

While this study provides valuable gender comparison information regarding depression following TBI, limitations exist. The present study sample of fewer women (25%) than men (75%) may not be adequate to fully evaluate the gender differences in depression post-TBI; we should note, however, that this gender ratio is representative of individuals who have sustained a moderate to severe TBI across all TBI Model Systems throughout the U.S. (Frost et al., 2013; Model Systems Knowledge Translation Center, 2016) with female TBI representation being even smaller in the Veteran population (Armed Forces Health Surveillance Center, 2013). It is also important to highlight that individuals in this study sample were slightly younger and more racially and ethnically diverse, compared with the Model Systems National Database as a whole (Model Systems Knowledge Translation Center, 2016). Future research is needed to extend this study to assess depression in women and men with TBI across the country. Finally, we used data from only one time point, as opposed to measuring depression over a series of time points, thus limiting our ability to assess spontaneous symptom recovery, relapse, or a change in depression over time. We plan to expand this research by analyzing data from the entire Model Systems National Database, and to assess depression at multiple time points, in order to more fully evaluate depression in women and men following TBI.

## Author Contributions

LI is the last author and anchored and directed the project. She also actively wrote sections of the “Introduction” and “Discussion” as well as assisted in directing the data analysis. SL, first author collected the data, conceived of the clinical outcomes, wrote the majority of the manuscripts. All remaining authors contributed to both data interpretation and writing of the paper.

## Conflict of Interest Statement

The authors declare that the research was conducted in the absence of any commercial or financial relationships that could be construed as a potential conflict of interest.
